# Climate change impacts on worldwide ecological niche and invasive potential of *Sternochetus mangiferae*

**DOI:** 10.1002/ps.8465

**Published:** 2024-10-09

**Authors:** Owusu Fordjour Aidoo, George Correa Amaro, Philipe Guilherme Corcino Souza, Marcelo Coutinho Picanço, Kwaafo Akoto Awuah‐Mensah, Ricardo Siqueira da Silva

**Affiliations:** ^1^ Department of Entomology, College of Agricultural, Human, and Natural Resource Sciences Washington State University Pullman WA USA; ^2^ Department of Biological Sciences, School of Natural and Environmental Sciences University of Environment and Sustainable Development Somanya Ghana; ^3^ Embrapa Roraima Boa Vista Brazil; ^4^ Instituto Federal de Ciência e Tecnologia do Triângulo Mineiro (IFTM Campus Uberlândia) Uberlândia MG Brazil; ^5^ Department of Entomology Universidade Federal de Viçosa Viçosa MG Brazil; ^6^ Department of Physical and Mathematical Sciences, School of Natural and Environmental Sciences University of Environment and Sustainable Development Somanya Ghana; ^7^ Department of Agronomy, Universidade Federal dos Vales do Jequitinhonha e Mucuri (UFVJM) Diamantina MG Brazil; ^8^ Department of Ecological Modelling Helmholtz Centre for Environmental Research—UFZ Leipzig Leipzig Germany

**Keywords:** species distribution modeling, MaxEnt, mango stone weevil, climate change

## Abstract

**BACKGROUND:**

Present climate studies on invasive species imply that climate change will alter the habitat suitability of invasive pests, especially given the projected rise in average global temperatures by the end of 2100. However, globally, limited information exists on the habitat suitability of the mango stone weevil, *Sternochetus mangiferae* Fabricius, which impedes the development of early detection and preventive measures. Herein, we used the MaxEnt model to estimate the potential global geographical distribution of *S. mangiferae*.

**RESULTS:**

Our results revealed that thermal conditions played a significant role in explaining the invasion risk of *S. mangiferae*. Habitat suitability was found in all continents, except Antarctica. Under the present condition, habitat suitability covered 5.67 × 10^7^ km^2^. For ssp126, habitat suitability will decrease from the 2060s (5.58 × 10^7^ km^2^) and 2080s (5.57 × 10^7^ km^2^). Similarly, under ssp585, suitable areas will decrease from 5.62 × 10^7^ to 5.51 × 10^7^ km^2^ for the 2060s and 2080s, respectively.

**CONCLUSION:**

Our study has estimated variability in the habitat suitability of *S. mangiferae* which establishes a foundation for determining global risk assessment and response plans for the pest. This study also identifies areas where the pest is inherently more vulnerable to the impacts of changing climates and enables forecasting of its potential distribution in a dynamic world. © 2024 The Author(s). *Pest Management Science* published by John Wiley & Sons Ltd on behalf of Society of Chemical Industry.

## Graphical abstract

We defined the global climate suitable areas for mango stone weevil using the MaxEnt model. The findings provide a theoretical foundation for biosecurity and risk assessment.

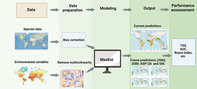

## 1 INTRODUCTION

Global warming affects the ecological range of insects by causing these species to migrate into new areas. Such warming temperatures can profoundly influence insects' development, distribution, and phenology in agriculture, affecting crop production and food security.^1^ Recent studies have demonstrated that temperature changes alter the geographical distribution of invasive species.^2^, ^3^, ^4^ The spread of invasive species has been facilitated by factors, including forest sector activities, economics, travel, trade, tourism, and regulatory regimes.^5^, ^6^, ^7^, ^8^ Invasive species have received global attention because their impacts are compounded by climate change and global warming.^9^ Therefore, understanding the potential distribution of invasive species like the mango stone weevil (MSW) *Sternochetus mangiferae* Fabricius (Coleoptera: Cuculidae) is paramount to its biological invasion as such modeling results can provide a theoretical framework for policy formulation and development of plant protection and regulatory plans.

MSW was first discovered in India but has rapidly spread due to globalization and international trade. It is currently distributed in Africa, Asia, Australia, the Caribbean, and the Pacific islands.^10^ The MSW is classified as a damaging quarantine pest by the Caribbean Plant Protection Commission, Inter‐African Phytosanitary Council, North American Plant Protection Organization, and Organismo Internacional Regional de Sanidad Agropecuaria.^11^ MSW overwinters under the loose bark near the base of mango trees and in the forks of branches but can also live in leaf litter around the tree. Adults of MSW can survive for about 3 years without a host.^12^ Balock and Kozuma^13^ reported that the onset of diapause was associated with long‐day photoperiod and stopped during a short‐day photoperiod.

The host range of the MSW is confined to mangoes and feeds on the leaves, young shoots, and flower buds. Females of MSW start laying eggs within 3–4 days after mating on different sizes of mango fruits, ranging from marble‐sized to fully unripened fruit‐sized.^14^ However, eggs are usually laid on the sinus of the fruits or stems.^15^ The oviposition periods vary, ranging from 3 to 6 weeks.^15^, ^16^ A female can lay about 15 eggs daily, with about 300 eggs in 3 months under laboratory conditions.^13^ The egg incubation lasts about 5 to 7 days, depending on seasonal variations and temperature conditions.^13^ After hatching, the MSW larva burrows into the seed through the mango fruit.^13^ There are five to seven larval stages, and the complete larval development varies across different locations. For instance, complete larval development takes about a month in southern India,^14^ 22 days to 10 weeks,^14^, ^17^ and about 40 days in the Northern Territory of Australia.^15^ The pupae stage occurs in the seed and is rarely found in the flesh. The pupa stage lasts about a week.^14^ The development time from egg to adult has been estimated at 35–54 days in India,^14^, ^18^ whereas 45–58 days have been reported in Australia,^15^ suggesting environmental factors play a role in its development.

The economic impact of the MSW is principally assessed based on its phytosanitary consideration, thereby restricting access to new international markets and contributing to rejections of fruit bound for existing export countries.^19^ MSW is a significant concern to the mango industry in Africa because the crop serves as a food source for domestic and export markets. For the latter, the presence of MSW in the mango production chain justifies the export rejection of mango fruits.^20^, ^21^ Schotman^22^ reported that the presence of weevils does not have an unfavorable effect on mango fruit growth, but the mere presence of weevils in fruits can result in shipments being rejected for export. MSW infestation and premature fruit drop have been estimated to be between 5% and 80%.^23^

MSW management and control strategies include quarantine measures, farm sanitation, and mechanical, physical, biological, and chemical control. The latter appears to be the most common management strategy by mango farmers.^24^ However, concerns over chemical residues in mango fruits and resistance development in MSW populations^19^, ^25^ suggest that a more friendly approach is needed to manage MSW in mango plantations. In response, several studies have used natural enemies, such as the African Weaver Ant *Oecophylla longinoda* (Latreille),^26^ entomopathogens *Beauveria bassiana* ^27^ to reduce field populations of MSW. Despite the efforts to control MSW using biological control agents, the pest continues to affect foreign local markets of many mango‐producing countries. Therefore, a clear understanding of its potential distribution can contribute to developing surveillance, monitoring, and prevention programs.

Species distribution models (SDMs) are a group of techniques that combine species occurrence data with environmental data to predict the ecological needs of the species by employing machine learning algorithms.^28^ These models are widely used in ecological and biogeographic studies to assess the potential distribution of species at different times and spaces to identify the areas that are suitable for the species.^29^, ^30^ SDMs have been widely used to assess the invasive potential of species.^2^ Such models have been used to predict the potential distribution of species, both at the regional and global scales. Among the SDMs, a machine learning algorithm based on the maximum entropy model has been widely used because it works well with small sample sizes and performs well when absent data are unavailable. Da Silva *et al*.^31^ assessed the habitat suitability of MSW in different Brazilian states using the MaxEnt model. However, to the best of our knowledge, no study has evaluated the habitat suitability of the pest, globally.

In this study, we predicted the potential global distribution of MSW using an updated global occurrence record with the MaxNet package in R statistical software to fit the MaxEnt model and identify climate‐suitable areas for the pest. It was also necessary to analyze the changes in suitable areas for the pest under current and future climate change scenarios [shared socioeconomic pathways (SSPs) (SSP126 and SSP585)] for the 2060s and 2080s. The results from this study provide a theoretical basis and data support for surveillance, monitoring, and development of ecologically friendly methods.

## 2 MATERIALS AND METHODS

**Figure 1:**
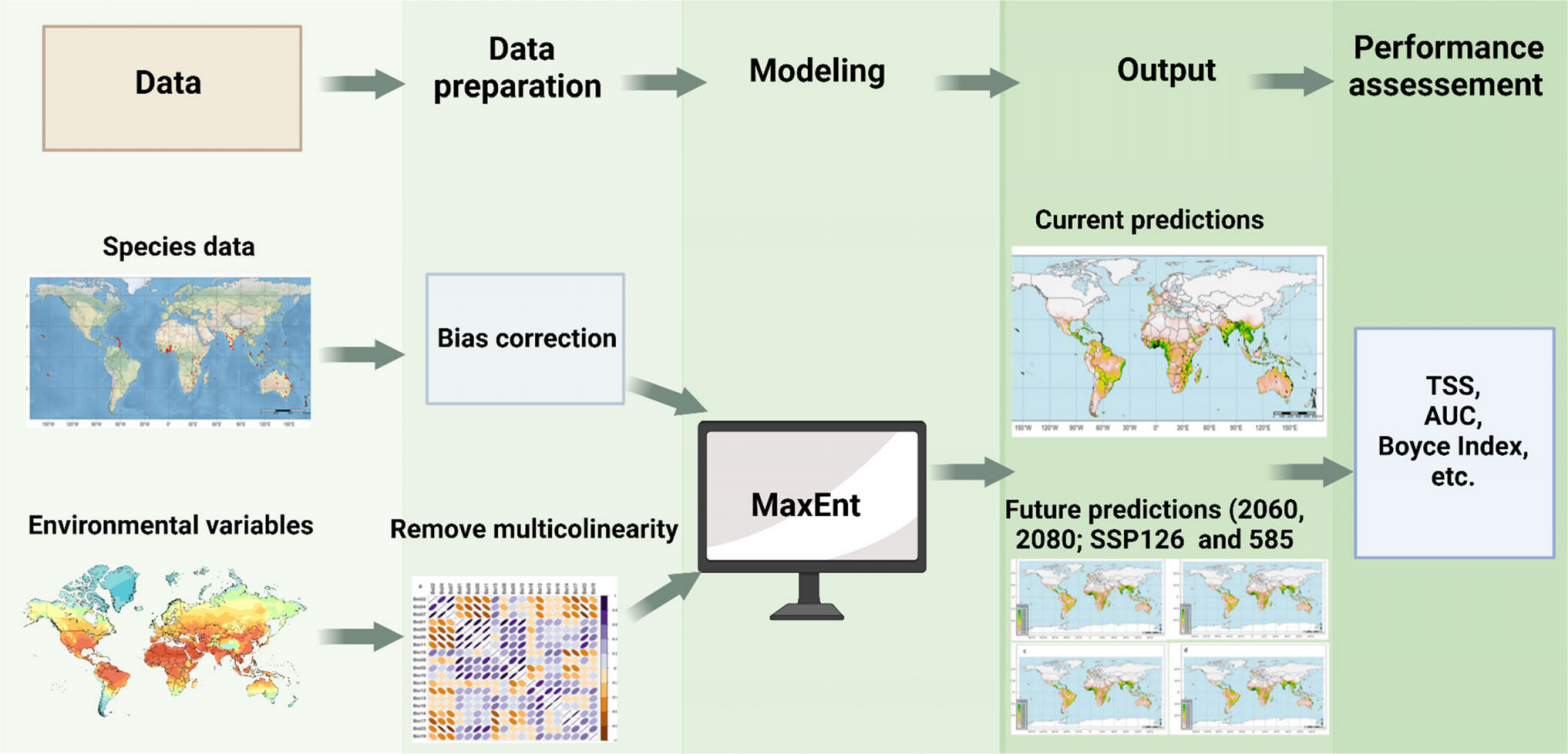
Technical flow chart of the study.

All procedures related to data processing, model development, and map creation were conducted in the R environment (version 4.3.0).^32^ The modeling process was divided into four main stages: (i) obtaining species data and cleaning; (ii) obtaining and selecting environmental variables; (iii) MaxEnt predictions; and (iv) creating habitat suitability maps for MSW. We illustrate the technical flow chart of the study in Fig. 1.

### 2.1 Occurrence data

The global occurrence records of MSW were obtained from scientific literature,^24^, ^33^, ^34^, ^35^, ^36^, ^37^ and supplemented by data from online databases, namely Centre for Agriculture and Bioscience International (CABI: https://www.cabi.org; Plantwise Knowledge Bank: https://plantwiseplusknowledgebank.org, and Global Biodiversity Information Facility (GBIF): https://www.gbif.org). The search criteria yielded 253 occurrence points. The data were filtered out to remove duplicates and erroneous records using the ‘flexsdm’ package and enforcing a 5 km distance to avoid sampling bias.^38^, ^39^ After this procedure, 16 occurrence records of MSW (6.32%) out of the total 253 points initially considered were found to be duplicates and were removed (Supporting Information Table S1). Following these steps, 237 occurrences were considered for the modeling process (Supporting Information Fig. S1(a),(b)).

### 2.2 Environmental data

**Figure 2:**
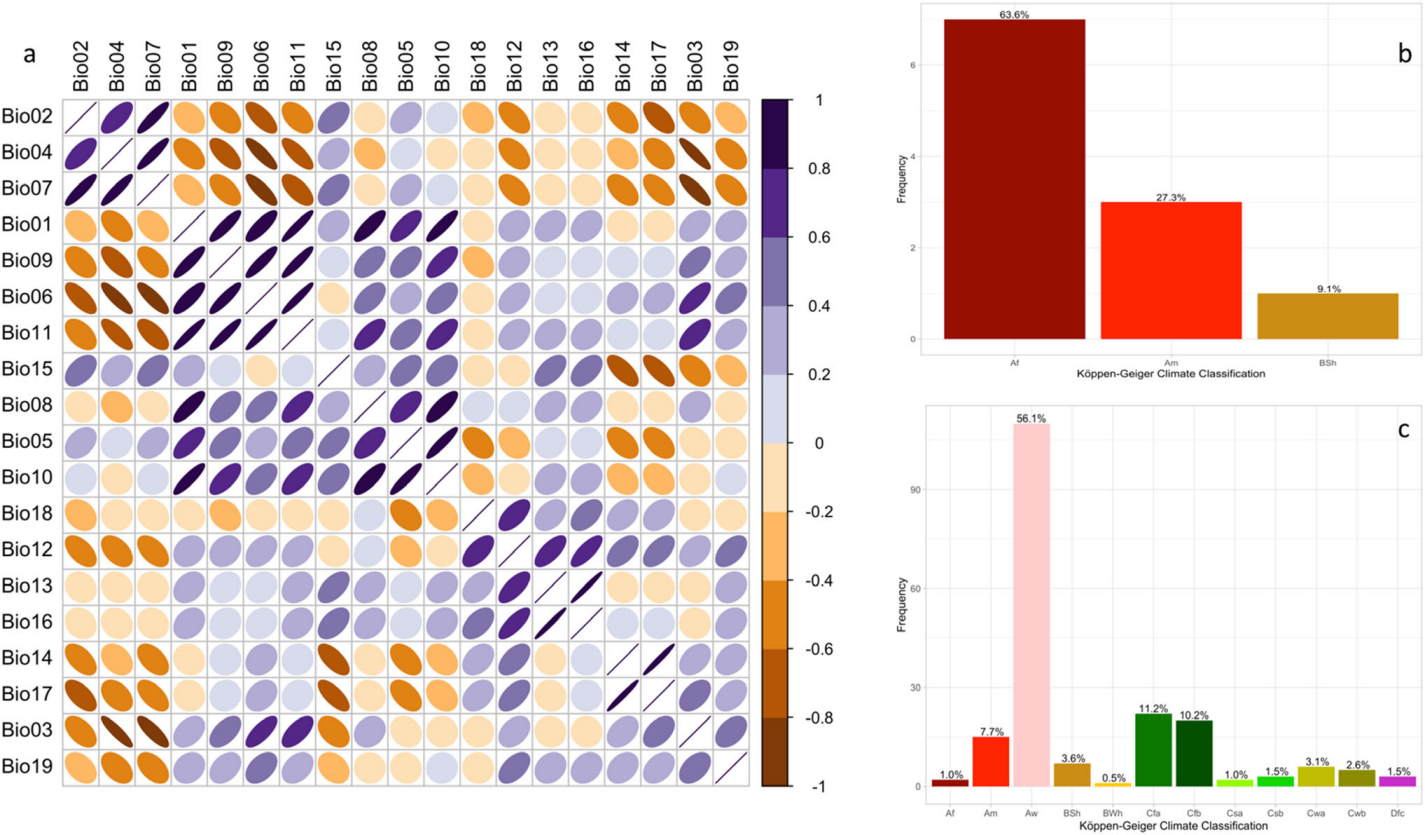
(a) Relationships between bioclimatic factors. Correlations are positive when the blue color slopes to the right and negative when the red color slopes to the left. Beginning at zero on the circle and progressing through the intermediate ellipse and line, the strength of Pearson's correlation coefficient (*r*) grows. Centroids were used in hierarchical cluster analysis to group variables with a correlation coefficient greater than 0.70. (b, c) Frequency histograms of the climatic classes occupied by the recorded occurrences of *Sternochetus mangiferae*. Native (b) and Invasive (c) ranges. Bio1 (annual average temperature), Bio02 (mean diurnal range), Bio03 (isothermality), Bio04 (seasonality of temperature), Bio05 (highest temperature of the hottest month), Bio06 (lowest temperature of the coldest month), Bio07 (annual temperature variation), Bio08 (average temperature of the rainy quarter months), Bio09 (average temperature of the driest quarter months), Bio10 (average temperature of the hottest quarter months), Bio11 (average temperature of the coldest quarter months), Bio12 (annual precipitation), Bio13 (precipitation of the rainiest month), Bio14 (precipitation of the driest month), Bio15 (precipitation seasonality), Bio16 (precipitation of the rainiest quarter months), Bio17 (precipitation of the driest quarter months), Bio18 (precipitation of the hottest quarter months), and Bio19 (precipitation of the coldest quarter months); Tropical monsoon climate (Am), Tropical dry savanna climate (As), Tropical savanna, wet (Aw), Hot semi‐arid (steppe) climate (Bsh), hot deserts climate (BWh), cold desert climate (BWk), humid subtropical climate (Cfa), monsoon‐influenced humid subtropical climate (Cwa), subtropical highland climate or temperate oceanic climate with dry winters (Cwb), Tropical rainforest climate (Af), cold semi‐arid (steppe) climate (BSk), temperate oceanic climate (Cfb), hot‐summer Mediterranean climate (Csa), and warm‐summer Mediterranean climate (Csb).

The bioclimatic variables from 1970 to 2000 used in this study were obtained from the Worldclim database version 2.1 (Table S2) using the ‘geodata’ package.^40^, ^41^ These variables have an average spatial resolution of 2.5 arc‐min, approximately equal to 4 km at the equator. Next, we considered the 2041–2060, and 2061–2080 periods for the SSP126 and SSP585. The SSPs delineate distinct developmental trajectories, accounting for potential trends in radiative forcing (in W/m^2^).^42^ Under the SSP126 scenario, an SSP‐based concentration‐driven model shows minimal radiative forcing at the century's end. By 2100, the radiative forcing level will reach 2.6 W/m^2^, as this scenario closely tracks the representative concentration pathway (RCP) RCP2.6 global forcing trajectory under SSP1 socioeconomic conditions. In contrast, SSP585 envisions a society dependent on fossil fuels and intensive energy use, anticipating a radiative forcing of 8.5 W/m^2^ in 2100 and a global temperature rise between 3.5 and 5.5 °C.^42^, ^43^ To address multicollinearity among bioclimatic variables, variables with a correlation coefficient |*r*| > 0.70 were excluded (Fig. 2(a), Tables S3–S6). The ‘corrplot’ package was used for the cluster analysis.^44^

### 2.3 Model development, calibration, and evaluation

MaxEnt model, based on maximum entropy, was selected for its extensive use in species distribution modeling and demonstrated efficacy compared to alternative methods.^29^ MaxEnt has been applied for assessing suitable regions for invasive species establishment globally^2^ and is recognized as a dependable approach in species distribution modeling.^45^ However, MaxEnt is susceptible to sample bias and potential overfitting issues.^46^ To optimize the MaxEnt model, a species‐specific adjustment process was employed.^47^ This approach aimed to avoid excessive complexity, which could reduce performance when projecting the model to diverse locations or under various climate change scenarios.^48^, ^49^ We finetuned the MaxEnt settings based on linear (L), quadratic (Q), product (P), and hinge (H).^50^, ^51^ One hundred and nineteen models (FC = L, Q, H, LQ, QH, LQH, LQP, LQHP; RM = 1 to 5, with increments of 0.25) were fitted to determine the best combination. Finally, the best model with RM = 0.5 and FC = LQH was used for the model settings.

The calibration area for the model was determined by considering the Köeppen–Geiger zones occupied by the species,^52^, ^53^ as illustrated in Fig. S1(b). The resulting calibration area covered 68 026 420 km^2^. Utilizing this approach is relevant for models intended for extrapolation to different geographic regions beyond the calibration area or for alternative temporal intervals.^38^, ^54^ To select 10000 background points from the calibrated area, a k‐fold cross‐validation was used by dividing the occurrence points into four blocks. Thirty grids from each block, varying in resolution from 0.5° to 8°, were generated, each ensuring a minimum of ten occurrences per partition (Fig. S2(a),(b)). This method effectively addresses potential spatial autocorrelation challenges between training and test data, offering a more suitable evaluation of model transferability than alternative partitioning methods.^55^, ^56^ Lastly, we determined the contribution of the environmental variables using the ‘varImportance’ function within the ‘fit MaxNet’ package.

The receiver operating characteristic (ROC) curve is independent of threshold and scale and has been extensively utilized for assessing the performance of SDMs.^49^ According to Hosmer *et al*.,^57^ a ROC curve boasting an area under the ROC curve (AUC) value of 0.9 or higher signifies an exceptional model fit, while a value falling between 0.7 and 0.9 is deemed reasonable. Conversely, a value of 0.5 or lower implies that the model performs no better than random chance. The positioning of the true positive rate (TPR) near 1 signifies an elevated level of sensitivity. The true skill statistic (TSS) is a numerical measure within the 0 to 1 range, with a value exceeding 0.9 considered ideal and a range between 0.85 and 0.9 deemed exceptional. TSS values falling between 0.7 and 0.85 are classified as very good, while those between 0.5 and 0.7 are considered good. Moreover, a TSS value within the 0.4 to 0.5 range is considered decent, and any value less than or equal to 0.4 indicates a poor fit.^58^

## 3 RESULTS

According to the TSS metric (= 0.59587), the optimal configuration of the MSW model was achieved by utilizing the linear, quadratic, and hinge (LQH) classes concurrently with a regularization multiplier of 0.5. The assessment metrics for the chosen model out of the 50 models tested, supplied by the ‘flexsdm’ package and computed from these models, are detailed in Table S7, with the models exhibiting superior performance compared to the random baseline.

The ROC curve of the final model (Fig. S2), derived from assessing true positive predictions (sensitivity) and false positive predictions (1 − specificity), demonstrated a valuable predictive capability with an AUC value ranging between 0.8 and 0.9 (Fig. S2(a)). Figure S2(b) precisely depicts the ROC curve and partial AUC details when constraining the false positive rate (FPR) and TPR (*x* = FPR = specificity; *y* = TPR = sensitivity) within the 90–100% range. The partial area (pAUC) can be interpreted as the average sensitivity within the specified specificity range and the average specificity within the specified sensitivity range.

The response curves of the model are depicted in Fig. S3, offering insights into the average marginal impact of environmental variables on the suitability of the environment for MSW. These graphs elucidate how each predictor variable influences the model response individually while maintaining the effects of the other variables constant. The most favorable habitats (optimal values) for MSW, as predicted by our model, are detailed in Table S8. The response curves for the training and projection data are also presented in Fig. S4. Additionally, the histogram illustrating the occurrence of MSW concerning environmental variables is presented in Fig. S5.

In its native regions, MSW primarily inhabits areas characterized by a tropical rainforest climate (Af), tropical monsoon climate (Am), and hot semi‐arid (steppe) climate, as per the updated Köppen–Geiger climate classification **(**Fig. 2(b) **)**. Approximately 100% of the data points fall within these climate classes. However, in the invaded regions, the occurrences are more concentrated (around 77%) in the climate classes of tropical savanna, wet (Aw), humid subtropical climate (Cfa), and temperate oceanic climate (Cfb) (Fig. 2(b)). Notably, we observed a broader range of climate classes occupied by MSW in newly invaded areas compared to its native regions, indicating a shift in its ecological niche.

**Figure 3:**
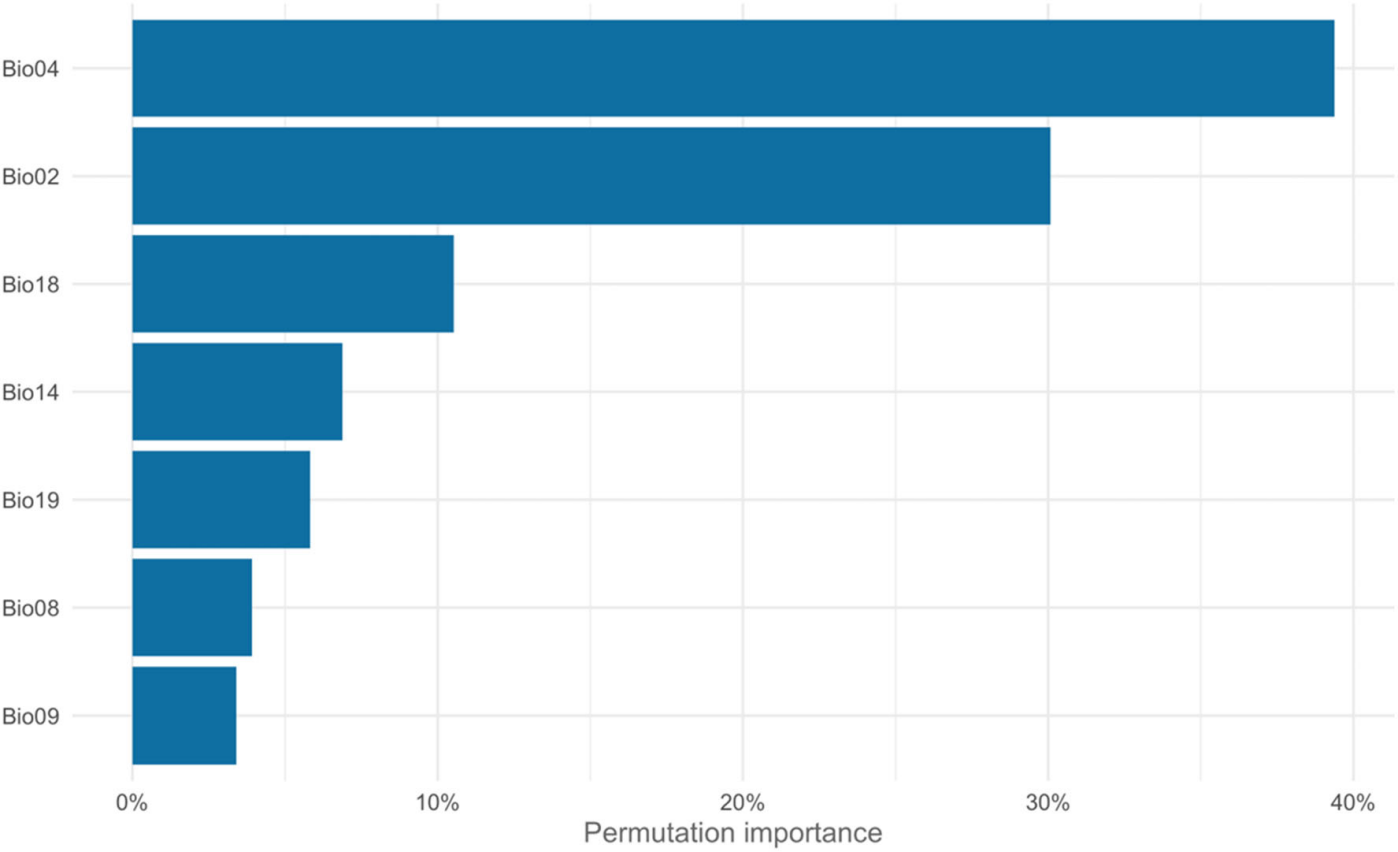
Percentage contribution of environmental variables to the final model of *Sternochetus mangiferae*. Mean diurnal range (Bio02), temperature seasonality (Bio04), mean temperature of wettest quarter (Bio08), mean temperature of driest quarter (Bio09), precipitation of driest month (Bio14), precipitation of warmest quarter (Bio18), precipitation of coldest quarter (Bio19).

The analysis revealed the hierarchy of significance among the seven bioclimatic variables, with temperature seasonality (Bio04) > average variation of daytime temperature (Bio02) > precipitation of the hottest quarter months (Bio18) > precipitation of the driest month (Bio14) > precipitation of the coldest quarter (Bio19) > average temperature of the rainy quarter months (Bio08) > average temperature of the driest quarter months (Bio09) emerging as the most influential factors driving the distribution of MSW. Among these environmental variables, Bio04, Bio02, Bio18, Bio19, Bio08, and Bio09 collectively contributed to approximately 95% of the model's explanatory power **(**Fig. 3 **)**.

**Figure 4:**
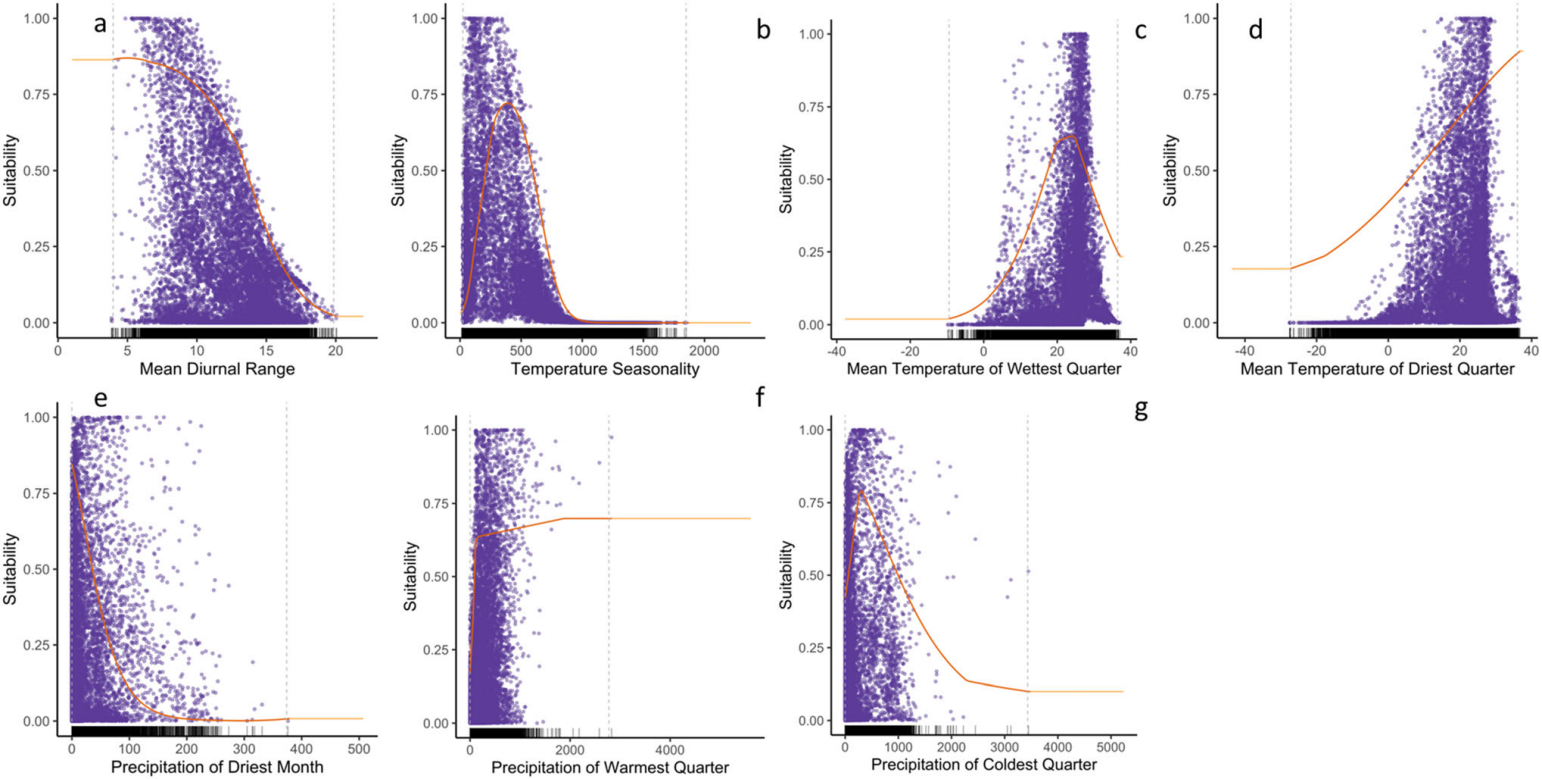
Histogram depicting the occurrence of *Sternochetus mangiferae* concerning environmental variables. (a) Mean diurnal range (Bio02), (b) temperature seasonality (Bio04), (c) mean temperature of wettest quarter (Bio08), (d) mean temperature of driest quarter (Bio09), (e) precipitation of driest month (Bio14), (f) precipitation of warmest quarter (Bio18), and (g) precipitation of coldest quarter (Bio19).

One way to see how the occurrence records are distributed is through a partial dependency plot. The results showed that MSW was more likely to be found in areas with a high probability of occurrence points as predicted by the model **(**Fig. 4 **)**. Tukey's analysis showed a significant difference between the current and future predictions (Fig. S6). However, no significant difference was detected for SSP126 in the 2060s and 2080s (Fig. S6). Similarly, the analysis showed no significant relationship between the 2060s and 2080s under the SSP585 climate change scenario.

**Figure 5:**
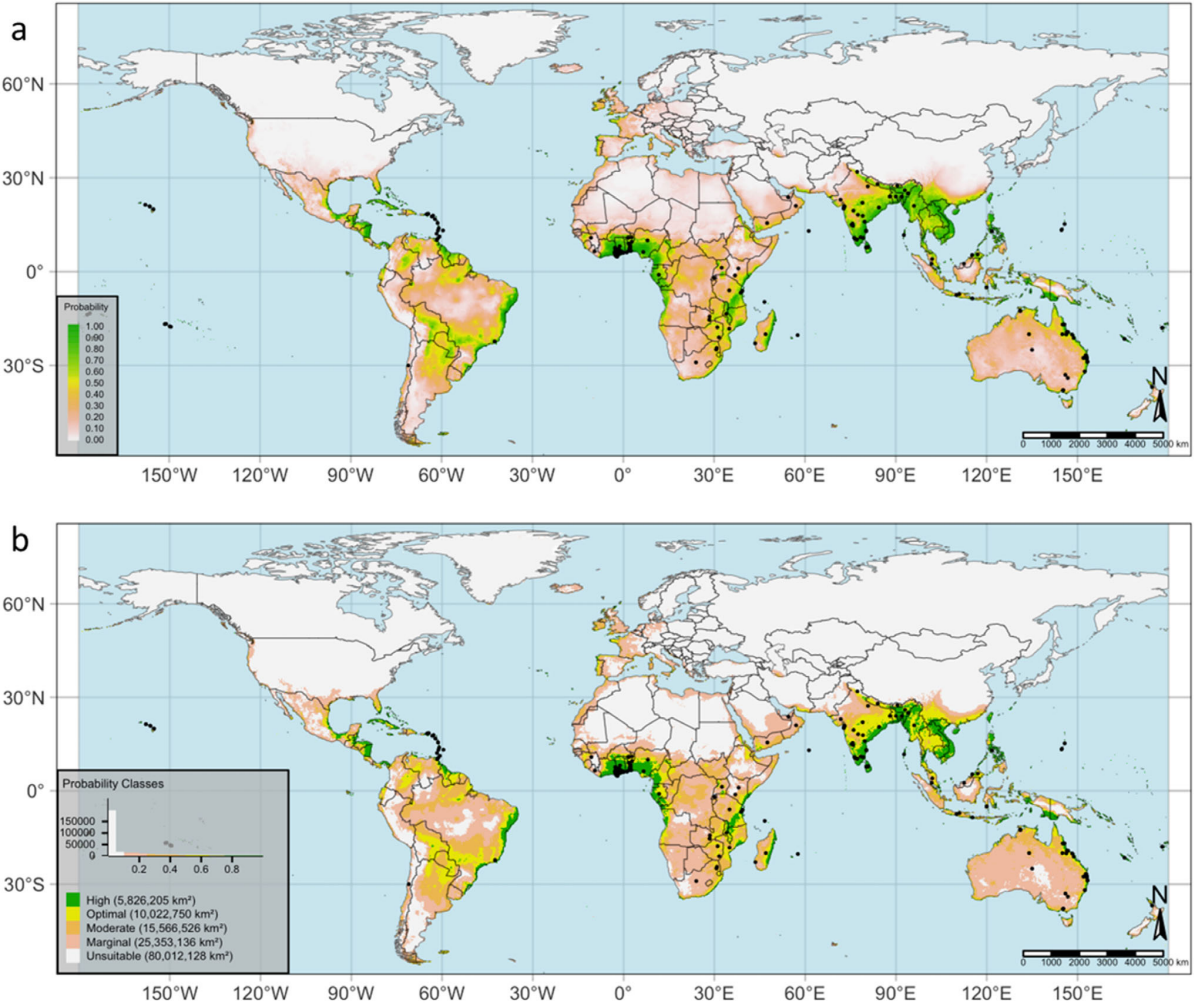
(a) Current global potential habitat suitability of *Sternochetus mangiferae* based on (a) the probability of occurrence and (b) MaxEnt classes.

As generated by our model, the predicted potential global geographic distribution of MSW under the current time is depicted in Fig. 5(a), presenting the probability of establishment categorized into seven classes to facilitate visualization and comparison across different locations. The calculated probabilities for high, optimal, moderate, marginal, and unsuitable conditions were 100% (Table S9, Fig. 5(b)). Applying a threshold that maximizes the sum of sensitivity and specificity (max_sens_spec = 0.3656577) yielded the map shown in Fig. 5(b), covering an area of 5 826 205 km^2^ (Table S9). The model predicts the expansion of climate‐suitable areas from the currently known occurrence records of the pest. Based on the current prediction, the newly identified areas include parts of Spain, Portugal, Sweden, and Italy in Europe; Mexico and the United States in North America; Argentina, Uruguay, and Paraguay in South America; northern fringes of Africa; Namibia, Angola, Mali, and Democratic Republic of Congo in Africa; Papua New Guinea in Oceania; and China, and Pakistan in Asia **(**Fig. 5(a),(b) **)**.

**Figure 6:**
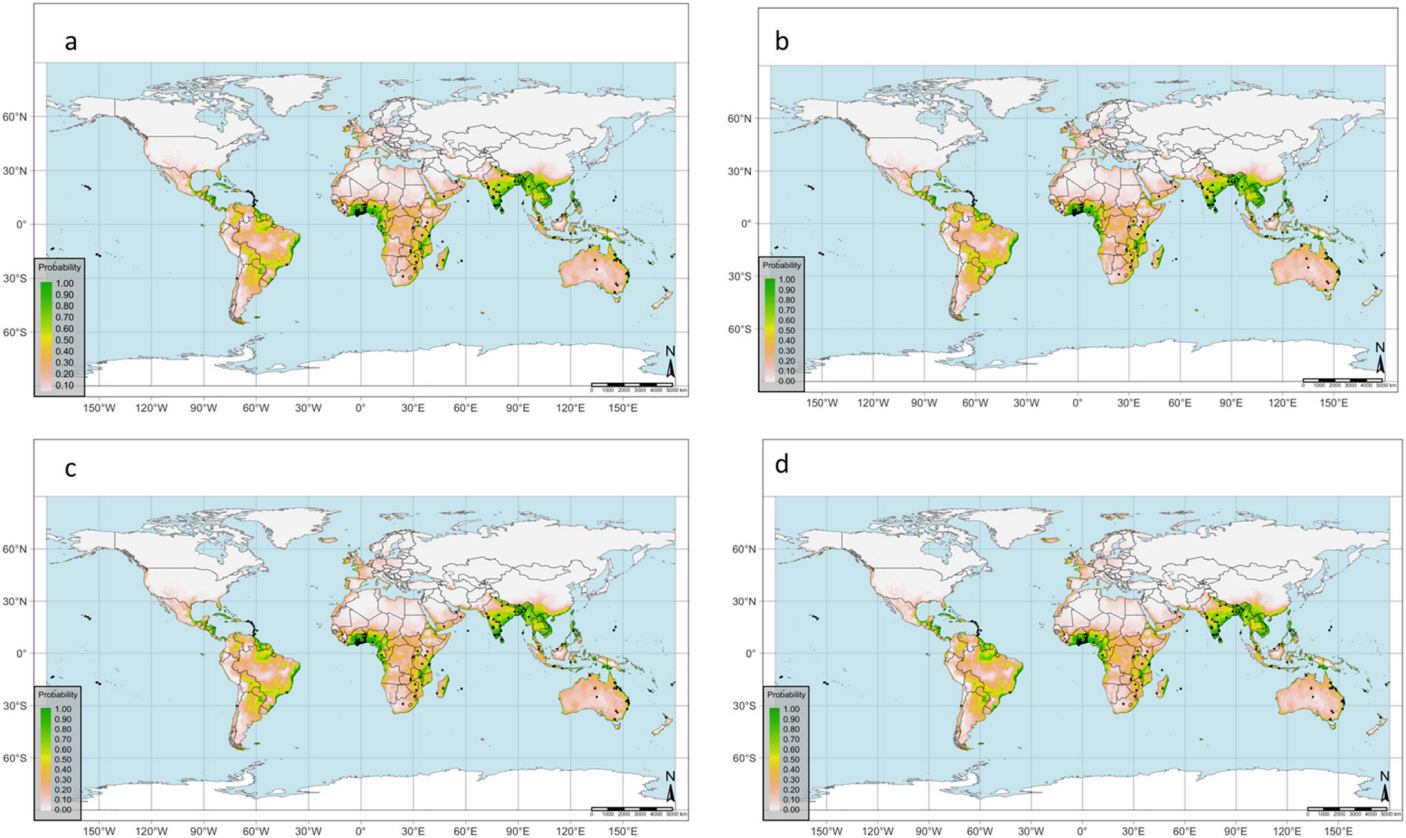
Predicted habitat suitability of *Sternochetus mangiferae* based on the probability of occurrence for (a) 2060s and (b) 2080s for SSP126 and (c) 2060s and (d) 2080s for SSP585.

**Figure 7:**
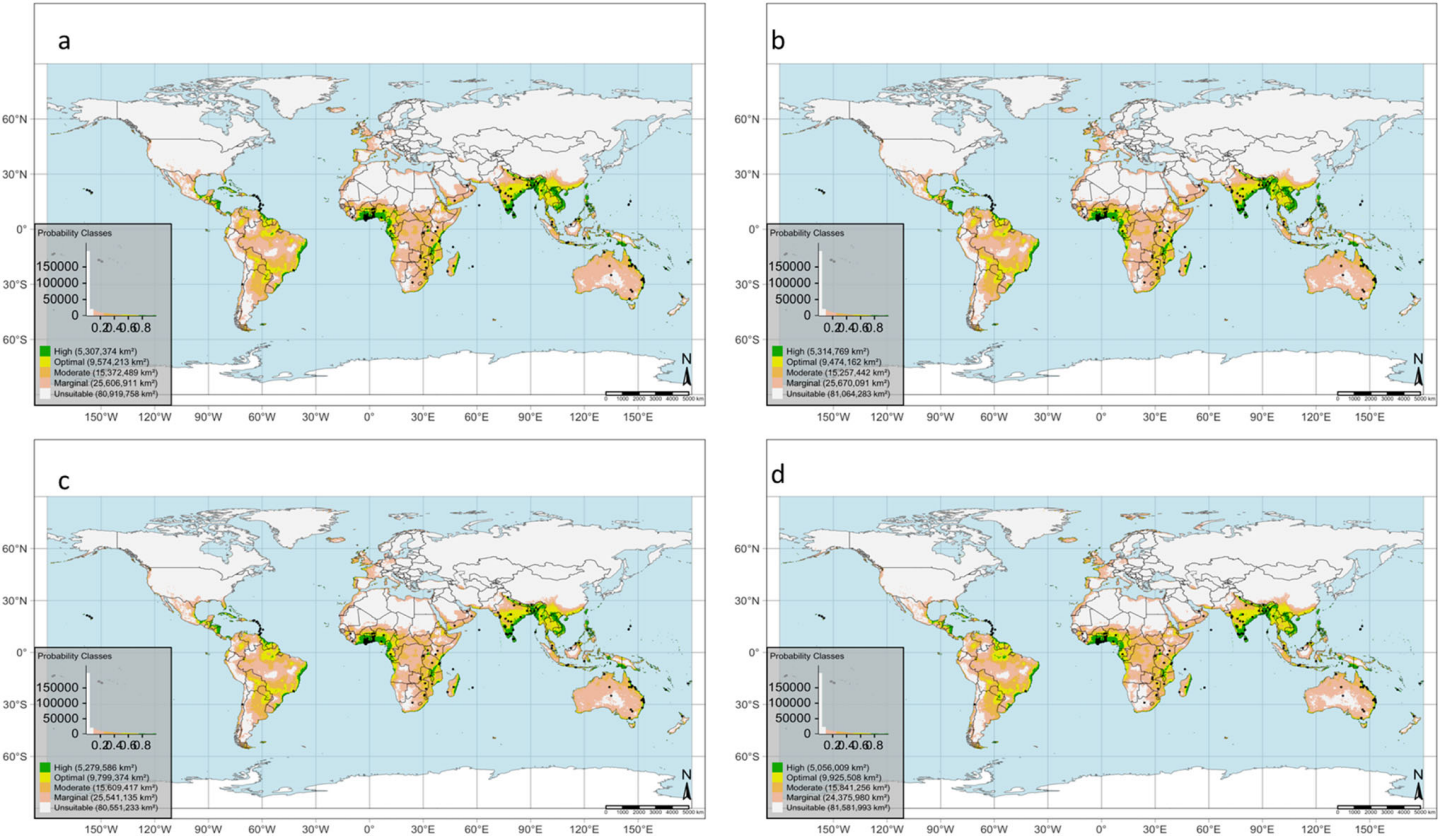
Predicted future habitat suitability of *Sternochetus mangiferae* based on the MaxEnt world classes and computed climate‐suitable areas of the pest for (a) 2060s and (b) 2080s for SSP126 and (c) 2060s and (d) 2080s for SSP585.

The projected distribution regions for MSW worldwide under the SSP126 and SSP585 and for the two specified periods (the 2060s and 2080s) are depicted in Fig. 6 and detailed in Table S9. The model's predicted suitability classes for the country are visualized in Fig. 7, encompassing marginal to highly suitable habitats. However, areas exhibiting high suitability are predominantly situated in the southern regions of the predicted areas. For SSP126, the suitability will decrease from the 2060s (5.58 × 10^7^ km^2^) and 2080s (5.57 × 10^7^ km^2^). Similarly, suitable areas will decrease from 5.62 × 10^7^ to 5.51 × 10^7^ km^2^ for the 2060s and 2080s under SSP585, respectively (Table S9 and Fig. 7). Similarly, unsuitable climate areas will increase from the 2060s (8.05 × 10^7^ km^2^) to 2080s (8.15 × 10^7^ km^2^). Based on the modeling results, mango‐producing regions like Brazil, China, India, Indonesia, Mexico, and Pakistan are projected to undergo changes in suitable climate areas from the current period to the future, with most of these countries showing high climate suitability for MSW in the future **(**Figs 6 and 7 **)**.

## 4 DISCUSSION

The MSW, a significant pest of mango, is quickly spreading across all continents. It is critical to assess the spread of the MSW due to its impacts on global food security. In the present study, we fitted the MaxEnt model with the MaxNet package in R to estimate climate change impacts on the habitat suitability of the MSW. Our model demonstrates a substantial confidence level, as its performance surpasses random outcomes, indicating a strong agreement between the presence of MSW and its predicted habitat suitability. The present study showed that temperature seasonality, mean diurnal range, mean temperature of the driest quarter, precipitation of the driest month, precipitation of the warmest quarter, and precipitation of the coldest quarter were the most important variables affecting the global distribution of MSW. In contrast, da Silva *et al*.^31^ found mean annual temperature, annual precipitation, mean daytime temperature range, and annual temperature range to be the most important variables influencing MSW habitat suitability. Yet, both studies suggest that temperature, much more than rainfall variables, influences the pest's distribution. The effects of temperature on the biology of the MSW have been investigated, revealing that the pest's reproductive rate exhibits a non‐linear response to the recorded temperatures.^13^, ^59^ This observation underscores the sensitivity of MSW to temperature variation and provides evidence that the population growth and survival of this species could be negatively affected by climate change.^59^

The current study predicts climate‐suitable areas for MSW on every continent except Antarctica, covering global areas of 5.67 × 10^7^ km^2^. Conversely, da Silva *et al*.^31^ found no habitat suitability in Europe. Several factors, including the number of occurrence records and the calibrated area, can influence the model output. In the present study, we used 237 occurrences, whereas da Silva *et al*.^31^ used 64 occurrences of the MSW. Additionally, Amaro *et al*.^60^ demonstrated that the extent of the study area used for training the data can influence the model output. In the present study, the calibration was based on the Köppen–Geiger zones occupied by the species which is different from that of da Silva *et al*.^31^ Lastly, the present study used the MaxNet package in R to fit the MaxEnt model, whereas da Silva *et al*.^31^ used the MaxEnt Java software for their predictions, which could have influenced the prediction outcomes.

The current study's predictions covered mango‐producing countries in parts of Southern Asia, Southeast Asia, East and West Africa, the tropical and subtropical Americas, the Caribbean, and the province of Málaga, Spain.^61^, ^62^ In Spain, the EFSA Panel on Plant Health (PLH) report from 2018 showed that MSW had been eradicated. However, the predictions showed large areas had habitat suitability and if the pest were to invade and establish itself, it could significantly impact the Spanish economy. Therefore, our study proposes urgent measures to prevent the invasion of the pest in areas where it is currently absent but predicted to have habitat suitability. This study also suggests the need for regular monitoring and surveillance in these regions.

Our predictions show a reduction in marginal and highly climate‐suitable areas from the 2060s to the 2080s, accompanied by an increase in moderate and optimal climate‐suitable areas during the same period. Likewise, in the projected future scenario (SSP585), there is a decline in high and marginal suitable climate areas from the 2060s to the 2080s, coupled with an ascent in optimal and moderate areas during the same timeframe. Considering the current and future predictions, it can be concluded that MSW has the potential to threaten mango production until the 2080s. The most common means of MSW long‐distance dispersal are fruits and seeds, which can transport several stages of development, including larvae, pupae, and adults,^63^ which can facilitate its dispersal through international trade and movement of host plant materials.^64^, ^65^ As such, careful inspection of fruits for the pest at entry points of countries predicted to be suitable but without the pest could minimize potential invasion. Research on the frequency of outbreaks, irradiation of fruits,^66^ quarantine treatment,^14^ sanitation,^15^ biological control,^33^ hot and cold treatment,^14^ host plant resistance,^67^ and chemical‐based control methods^68^ can help sustainable management of the pest. Moreover, our study suggests that scientists and plant protection and regulatory services can use our MSW habitat suitability maps to choose which mango cultivars to plant carefully in specific areas.

The classification of the native and invaded areas suggests that those in the invaded areas have adapted to varying climates. Such invasive potential is worrying as MSW has been found in mango fruits and seeds traded internationally.^64^, ^65^ Moreover, with the rise in international trade, urgent attention is required for global phytosanitary regulation and the development of strategic measures using our predictive models to minimize the spread of MSW. With the help of our distribution maps, researchers can better understand the patterns of the MSW dispersion and pinpoint parts of countries where the MSW can be established. Officials can use our modeling outcomes to prioritize checking agricultural products entering or passing through areas with a higher risk of invasion, making it an essential tool for decision‐making. Moreover, this study adds to the existing body of knowledge by showing that the potential introduction of MSW will most likely occur in regions outside the presently known distribution areas.

The current study predicts the regions with habitat suitability for MSW invasion worldwide. The probability of its establishment was evaluated, and the outcomes were interpreted without accounting for certain constraints associated with the pest which should be considered in future studies. Our study employed bioclimatic climate variables, which are not the only factors influencing species distribution; our model does not consider biotic factors such as competition in the invaded areas, behavior and adaptation, and propagule pressure. The adult MSW has a weak dispersal capability; it can only fly short distances and usually stays near the host mango tree.^69^ Hence, anthropogenic activities are the primary vectors for the long‐distance spread of weevils,^21^, ^70^, ^71^ but were not considered. Moreover, MSW winter hiding places include the mango tree's loose bark, the forks in the branches, the leaf litter below the tree, and even the seeds of the mango tree.^70^ Farm‐level management practices like sanitation should have been considered in the present modeling. Furthermore, abiotic factors and government interventions for eradicating the pest, as in the case of Spain,^36^ were not considered. Farm‐level management strategies, early detection and surveillance policies, and rapid response programs after invasion can all influence the successful invasion and establishment of an invasive species like MSW. Future studies should include some of these factors in the development of MSW models. We did not consider natural and future factors that can influence the dispersal of the MSW as many invasive species are spread through human‐induced activities such as the movement of host plant materials. Future studies that incorporate the natural dispersal of the pest can provide more insights into its habitat suitability. We also encourage further research on this invasive pest and its socioeconomic effects.

Nonetheless, the resultant maps are considered reliable based on the acceptable range of the metrics and thresholds used for modeling the MSW. The predictions provide the theoretical basis of areas that can be used for setting traps thereby maximizing trap catches and reducing economic losses associated with monitoring and surveillance of the MSW.

## FUNDING INFORMATION

This work was funded by the Conselho Nacional de Desenvolvimento Cientifico e Tecnologico (CNPq), the Fundaçao de Amparo a Pesquisa do Estado de Minas Gerais (FAPEMIG), Empresa Brasileira de Pesquisa Agropecuaria (Embrapa) and the Coordenação de Aperfeiçoamento de Pessoal de Nível Superior – Brasil (CAPES) – Finance Code 001.

## CONFLICT OF INTEREST STATEMENT

The authors have no relevant financial or non‐financial interests to disclose.

## AUTHOR CONTRIBUTIONS

OFA conceived and designed the research. GCA and OFA conducted experiments and analyzed data. OFA and GCA wrote the manuscript with input from PGCS, MCP, KAA‐M, and RSS. All authors read and approved the manuscript.

## Supporting information


**Table S1.** Description of cleaning method, test name, numbers flagged and percentage number of *Sternochetus mangiferae* records removed prior to analysis.
**Table S2.** Initial environmental variables utilized to model the distribution of *Sternochetus mangiferae*.
**Table S3.** Variable permutation importance of the base model for *Sternochetus mangiferae*.
**Table S4.** Variable permutation importance for the base variable selection for *Sternochetus mangiferae*.
**Table S5.** Variable permutation importance of the base reduced variable for *Sternochetus mangiferae*.
**Table S6.** Occurrence descriptive statistics of the model for *Sternochetus mangiferae*.
**Table S7.** Metrics and thresholds for the final model of *Sternochetus mangiferae*.
**Table S8.** Response curves optimum values for *Sternochetus mangiferae*.
**Table S9.** Future predictions and calculated areas for *Sternochetus mangiferae* predictions under current and future climate change scenarios.
**Figure S1.** Global maps showing distribution of *Sternochetus mangiferae*: (a) Global occurrence records of *Sternochetus mangiferae*; (b) map of the Köeppen–Geiger zones used to define the model calibration area, considering the current dispersion of *Sternochetus mangiferae*.
**Figure S2.** A receiver operating characteristic (ROC) curve for the potential global geographic distribution model of *Sternochetus mangiferae*, showing the total area under the ROC curve (AUC) (a) and partial areas (pAUC) (b).
**Figure S3.** Response curves of the model. (a) Bio02 (mean diurnal range), (b) Bio04 (temperature seasonality), (c) Bio08 (average temperature of the rainy quarter months), (d) Bio09 (average temperature of the driest quarter months), (e) Bio14 (precipitation of the driest month), (f) Bio18 (precipitation of the hottest quarter months), and (g) Bio19 (precipitation of the coldest quarter months).
**Figure S4.** Partial dependence of climatic variables and occurrence of *Sternochetus mangiferae*; (a) Bio02 (mean diurnal range), (b) Bio04 (temperature seasonality), (c) Bio08 (average temperature of the rainy quarter months), (d) Bio09 (average temperature of the driest quarter months), (e) Bio14 (precipitation of the driest month), (f) Bio18 (precipitation of the hottest quarter months), and (g) Bio19 (precipitation of the coldest quarter months).
**Figure S5.** Histogram and density of occurrence of *Sternochetus mangiferae*. (a) Bio02 (mean diurnal range), (b) Bio04 (temperature seasonality), (c) Bio08 (average temperature of the rainy quarter months), (d) Bio09 (average temperature of the driest quarter months), (e) Bio14 (precipitation of the driest month), (f) Bio18 (precipitation of the hottest quarter months), and (g) Bio19 (precipitation of the coldest quarter months).
**Figure S6.** Tukey climates from the current time (1970–2000) to the future (2041–2060; 2061–2080) under SSPs 126 and 585 for the *Sternochetus mangiferae*.

## Data Availability

The data that support the findings of this study are available on request from the corresponding author. The data are not publicly available due to privacy or ethical restrictions.
