# Supplementary material for: Climate change impacts on worldwide ecological niche and invasive potential of Sternochetus mangiferae
Source: Pest Manag Sci. 2024 Oct 9;81(2):667–77. doi: 10.1002/ps.8465 (PMC11716358; doi:10.1002/ps.8465)
Supplement: Supplementary file 1 — Table S1. Description of cleaning method, test name, numbers flagged and percentage number of Sternochetus mangiferae records removed prior to analysis. Table S2. Initial environmental variables utilized to model the distribution of Sternochetus mangiferae. Table S3. Variable permutation importance of the base model for Sternochetus mangiferae. Table S4. Variable permutation importance for the base variable selection for Sternochetus mangiferae. Table S5. Variable permutation importance of the base reduced variable for Sternochetus mangiferae. Table S6. Occurrence descriptive statistics of the model for Sternochetus mangiferae. Table S7. Metrics and thresholds for the final model of Sternochetus mangiferae. Table S8. Response curves optimum values for Sternochetus mangiferae. Table S9. Future predictions and calculated areas for Sternochetus mangiferae predictions under current and future climate change scenarios. Figure S1. Global maps showing distribution of Sternochetus mangiferae: (a) Global occurrence records of Sternochetus mangiferae; (b) map of the Köeppen–Geiger zones used to define the model calibration area, considering the current dispersion of Sternochetus mangiferae. Figure S2. A receiver operating characteristic (ROC) curve for the potential global geographic distribution model of Sternochetus mangiferae, showing the total area under the ROC curve (AUC) (a) and partial areas (pAUC) (b). Figure S3. Response curves of the model. (a) Bio02 (mean diurnal range), (b) Bio04 (temperature seasonality), (c) Bio08 (average temperature of the rainy quarter months), (d) Bio09 (average temperature of the driest quarter months), (e) Bio14 (precipitation of the driest month), (f) Bio18 (precipitation of the hottest quarter months), and (g) Bio19 (precipitation of the coldest quarter months). Figure S4. Partial dependence of climatic variables and occurrence of Sternochetus mangiferae; (a) Bio02 (mean diurnal range), (b) Bio04 (temperature seasonality), (c) Bio08 (av [file PS-81-667-s001.docx]

Table S1. Description of cleaning method, test name, numbers flagged and percentage number of *Sternochetus mangiferae* records removed prior to analysis.

|  | Description | Test name | Records flagged | Percentage number records (*) |
| --- | --- | --- | --- | --- |
|  |  |  |  |  |
| 1 | Identical coordinates | .equ | 0 | 0 |
| 2 | Plain zeros | .zer | 0 | 0 |
| 3 | Records around the GBIF headquarters | .gbf | 0 | 0 |
| 4 | Duplicated coordinates per species | .dpl | 16 | 6.32 |
| 5 | Rounded (probably imprecise) coordinates | .rou | 0 | 0 |
| 6 |  |  |  |  |
| 7 | (*) calculated in relation to total number of records, i.e. 253 records |  |  |  |

**Table S2**. Initial environmental variables utilized to model the distribution of *Sternochetus mangiferae*.

| Code | Environmental variable | Unit |
| --- | --- | --- |
| Bio1 | Annual average temperature | °C |
| Bio2 | Mean diurnal range | °C |
| Bio3 | Isothermality | % |
| Bio4 | Seasonality of temperature | °C |
| Bio5 | Highest temperature of the hottest month | °C |
| Bio6 | Lowest temperature of the coldest month | °C |
| Bio7 | Annual temperature variation | °C |
| Bio8 | Average temperature of the rainy quarter months | °C |
| Bio9 | Average temperature of the driest quarter months | °C |
| Bio10 | Average temperature of the hottest quarter months | °C |
| Bio11 | Average temperature of the coldest quarter months | °C |
| Bio12 | Annual precipitation | mm |
| Bio13 | Precipitation of the rainiest month | mm |
| Bio14 | Precipitation of the driest month | mm |
| Bio15 | Precipitation seasonality | mm |
| Bio16 | Precipitation of the rainiest quarter months | mm |
| Bio17 | Precipitation of the driest quarter months | mm |
| Bio18 | Precipitation of the hottest quarter months | mm |
| Bio19 | Precipitation of the coldest quarter months | mm |

Table S3. Variable permutation importance of the base model for *Sternochetus mangiferae*.

| Variable | Permutation importance | Standard deviation |
| --- | --- | --- |
| Bio03 | 23.400 | 3.556 |
| Bio13 | 22.125 | 9.310 |
| Bio02 | 20.150 | 6.384 |
| Bio12 | 7.975 | 1.648 |
| Bio14 | 4.550 | 1.515 |
| Bio18 | 3.875 | 4.153 |
| Bio15 | 3.400 | 1.992 |
| Bio07 | 3.300 | 1.068 |
| Bio04 | 3.200 | 1.663 |
| Bio19 | 1.875 | 0.538 |
| Bio16 | 1.775 | 1.292 |
| Bio10 | 1.125 | 1.204 |
| Bio11 | 1.125 | 0.695 |
| Bio09 | 0.675 | 0.991 |
| Bio08 | 0.575 | 0.359 |
| Bio06 | 0.550 | 0.173 |
| Bio17 | 0.325 | 0.519 |
| Bio05 | 0.050 | 0.100 |
| Bio01 | 0.025 | 0.050 |

Table S4. Variable Permutation Importance for the base variable selection for *Sternochetus mangiferae*.

| Variable | Permutation importance | Standard deviation |
| --- | --- | --- |
| Bio04 | 37.000 | 3.873 |
| Bio02 | 27.400 | 5.611 |
| Bio18 | 10.025 | 5.326 |
| Bio14 | 9.875 | 0.645 |
| Bio19 | 6.125 | 1.434 |
| Bio08 | 4.025 | 2.464 |
| Bio09 | 3.225 | 3.272 |
| Bio15 | 1.425 | 0.377 |
| Bio05 | 0.875 | 0.723 |

Table S5. Variable permutation importance of the base reduced variable for *Sternochetus mangiferae*.

| Variable | Permutation importance | Standard deviation |
| --- | --- | --- |
| Bio04 | 39.375 | 2.737 |
| Bio02 | 30.075 | 4.990 |
| Bio18 | 10.525 | 5.934 |
| Bio14 | 6.875 | 0.818 |
| Bio19 | 5.825 | 1.406 |
| Bio08 | 3.925 | 1.692 |
| Bio09 | 3.400 | 2.202 |

Table S6. Occurrence descriptive statistics of the model for *Sternochetus mangiferae*.

| **Variable** | **Minimum** | **Maximum** | **Median** | **Mean** | **Standard deviation** |
| --- | --- | --- | --- | --- | --- |
| Bio02 | 5.29 | 17.40 | 8.87 | 9.44 | 2.36 |
| Bio04 | 33.30 | 658.06 | 154.07 | 213.13 | 141.93 |
| Bio08 | 11.07 | 30.85 | 26.18 | 25.29 | 3.07 |
| Bio09 | 7.32 | 32.62 | 24.87 | 23.27 | 4.79 |
| Bio14 | 0.00 | 183.00 | 20.00 | 27.53 | 30.80 |
| Bio18 | 0.00 | 1,627.00 | 339.00 | 383.76 | 236.43 |
| Bio19 | 0.00 | 1,976.00 | 257.00 | 257.23 | 269.31 |

Table S7. Metrics and thresholds for the final model of *Sternochetus mangiferae*.

| **Metric Names** | **Values** |
| --- | --- |
| True Positive Rate or Sensitivity (TPR) | 0.83798 |
| True Negative Rate or Specificity (TNR) | 0.75789 |
| True Skill Statistic (TSS) | 0.59587 |
| Sorensen Index | 0.08864 |
| Jaccard Index | 0.04668 |
| F-measure on Presence-Background (FPB) | 0.09335 |
| Omission or False Negative Rate (OR) | 0.16202 |
| Boyce Index | 0.87017 |
| Area Under ROC Curve (AUC) | 0.85162 |
| Inverse Mean Absolute Error (IMAE) | 0.78568 |
| Maximum Sensitivity plus Specificity (maxSSS) | 0.35115 |
| False Positive Rate (FPR) | 0.24211 |
| Positive Predictive Value or Precision (PPV) | 0.77584 |
| Accuracy | 0.79793 |
| F1 Score | 0.80571 |
| Balanced Accuracy | 0.79793 |
| Matthews Correlation Coefficient (MCC) | 0.59779 |
| Minimum Training Presence (MTP) | 0.06660 |
| 10th Percentile Training Presence (10TP) | 0.08425 |
| Symmetric Extremal Dependence Index (SEDI) | 0.55168 |

Table S8. Response curves optimum values for *Sternochetus mangiferae*

| Variable | Variable Name | Optimum Value | Probability |
| --- | --- | --- | --- |
| Bio02 | Mean Diurnal Range | 4.075667 | 1 |
| Bio04 | Temperature Seasonality | 326.7497 | 0.9822816 |
| Bio08 | Mean Temperature of Wettest Quarter | 22.52127 | 0.9697118 |
| Bio09 | Mean Temperature of Driest Quarter | 35.15107 | 0.9988562 |
| Bio14 | Precipitation of Driest Month | -18.3000000, -16.0818182, -13.8636364, -11.6454545, -9.4272727, -7.2090909, -4.9909091, -2.7727273, -0.5545455 | 0.9919499, 0.9919499, 0.9919499, 0.9919499, 0.9919499, 0.9919499, 0.9919499, 0.9919499, 0.9919499 |
| Bio18 | Precipitation of Warmest Quarter | 1789.7 | 0.9790531 |
| Bio19 | Precipitation of Coldest Quarter | 281.4303 | 0.9816196 |

Table S9. Future predictions and calculated areas for *Sternochetus mangiferae* predictions under current and future climate change scenarios.

| **Climate** | **Unsuitable (Km²)** | **Marginal (Km²)** | **Moderate (Km²)** | **Optimal (Km²)** | **High (Km²)** | **% Unsuitable** | **% Marginal** | **% Moderate** | **% Optimal** | **% High** |
| --- | --- | --- | --- | --- | --- | --- | --- | --- | --- | --- |
| **Currently (historic 1970-2000)** | **83,380,224** | **25,031,838** | **14,576,390** | **8,649,408** | **5,142,886** | **100.00** | **100.00** | **100.00** | **100.00** | **100.00** |
| CMIP6 BCC-CSM2-MR SSP126 (2041-2060) | 83,380,224 | 25,031,838 | 14,576,390 | 8,649,408 | 5,142,886 | 100.00 | 100.00 | 100.00 | 100.00 | 100.00 |
| CMIP6 CNRM-CM6-1 SSP126 (2041-2060) | 80,367,304 | 24,134,501 | 15,965,471 | 10,571,529 | 5,741,940 | 96.39 | 96.42 | 109.53 | 122.22 | 111.65 |
| CMIP6 MIROC6 SSP126 (2041-2060) | 80,762,089 | 25,908,924 | 14,943,740 | 9,633,596 | 5,532,397 | 96.86 | 103.50 | 102.52 | 111.38 | 107.57 |
| **CMIP6 Mean SSP126 (2041-2060)** | **80,919,758** | **25,606,911** | **15,372,489** | **9,574,213** | **5,307,374** | **97.05** | **102.30** | **105.46** | **110.69** | **103.20** |
| CMIP6 BCC-CSM2-MR SSP126 (2061-2080) | 83,044,483 | 25,003,075 | 14,601,311 | 8,725,042 | 5,406,834 | 99.60 | 99.89 | 100.17 | 100.87 | 105.13 |
| CMIP6 CNRM-CM6-1 SSP126 (2061-2080) | 80,987,872 | 24,200,240 | 15,581,052 | 10,373,270 | 5,638,311 | 97.13 | 96.68 | 106.89 | 119.93 | 109.63 |
| CMIP6 MIROC6 SSP126 (2061-2080) | 81,283,724 | 25,875,240 | 14,758,256 | 9,485,559 | 5,377,967 | 97.49 | 103.37 | 101.25 | 109.67 | 104.57 |
| **CMIP6 Mean SSP126 (2061-2080)** | **81,064,283** | **25,670,091** | **15,257,442** | **9,474,162** | **5,314,769** | **97.22** | **102.55** | **104.67** | **109.54** | **103.34** |
| CMIP6 BCC-CSM2-MR SSP585 (2041-2060) | 82,862,032 | 24,794,148 | 14,482,818 | 8,956,273 | 5,685,475 | 99.38 | 99.05 | 99.36 | 103.55 | 110.55 |
| CMIP6 CNRM-CM6-1 SSP585 (2041-2060) | 80,156,343 | 23,492,902 | 16,580,095 | 10,896,490 | 5,654,915 | 96.13 | 93.85 | 113.75 | 125.98 | 109.96 |
| CMIP6 MIROC6 SSP585 (2041-2060) | 81,332,820 | 26,003,281 | 14,280,641 | 9,836,060 | 5,327,945 | 97.54 | 103.88 | 97.97 | 113.72 | 103.60 |
| **CMIP6 Mean SSP585 (2041-2060)** | **80,551,233** | **25,541,135** | **15,609,417** | **9,799,374** | **5,279,586** | **96.61** | **102.03** | **107.09** | **113.30** | **102.66** |
| CMIP6 BCC-CSM2-MR SSP585 (2061-2080) | 84,666,531 | 22,614,974 | 14,139,213 | 9,389,588 | 5,970,440 | 101.54 | 90.34 | 97.00 | 108.56 | 116.09 |
| CMIP6 CNRM-CM6-1 SSP585 (2061-2080) | 81,199,262 | 22,058,049 | 16,982,754 | 10,885,370 | 5,655,312 | 97.38 | 88.12 | 116.51 | 125.85 | 109.96 |
| CMIP6 MIROC6 SSP585 (2061-2080) | 82,834,132 | 25,714,778 | 13,372,444 | 9,704,434 | 5,154,959 | 99.35 | 102.73 | 91.74 | 112.20 | 100.23 |
| **CMIP6 Mean SSP585 (2061-2080)** | **81,581,993** | **24,375,980** | **15,841,256** | **9,925,508** | **5,056,009** | **97.84** | **97.38** | **108.68** | **114.75** | **98.31** |


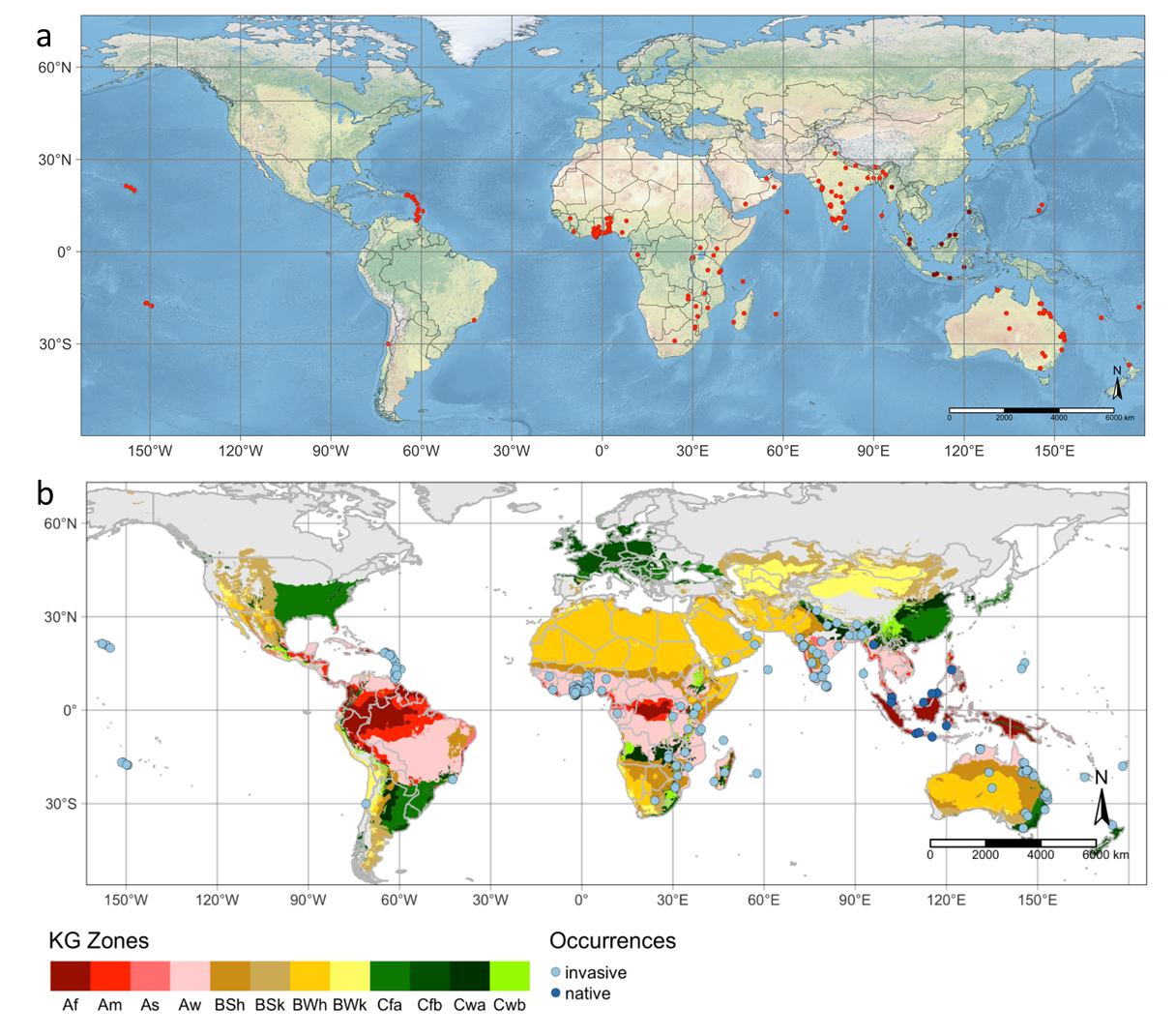


Figure S1. Global maps showing distribution of *Sternochetus mangiferae*: a) Global occurrence records of *Sternochetus mangiferae*; and b) Map of the Köeppen-Geiger zones used to define the model calibration area, considering the current dispersion of *Sternochetus mangiferae*,


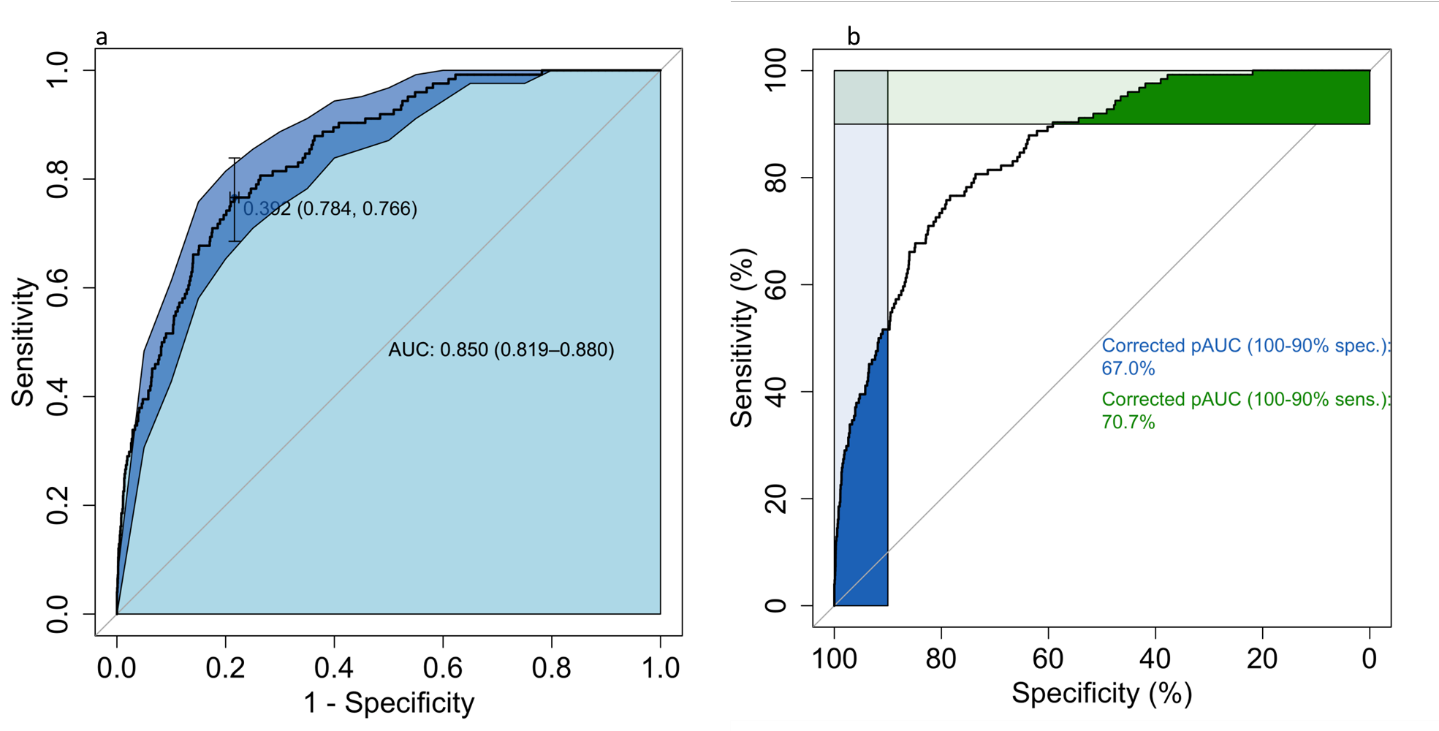


Figure S2. A Receiver operating characteristic (ROC) curve for the potential global geographic distribution model of *Sternochetus mangiferae*, showing the total area (AUC) under the ROC curve (a) and partial areas (pAUC) (b).


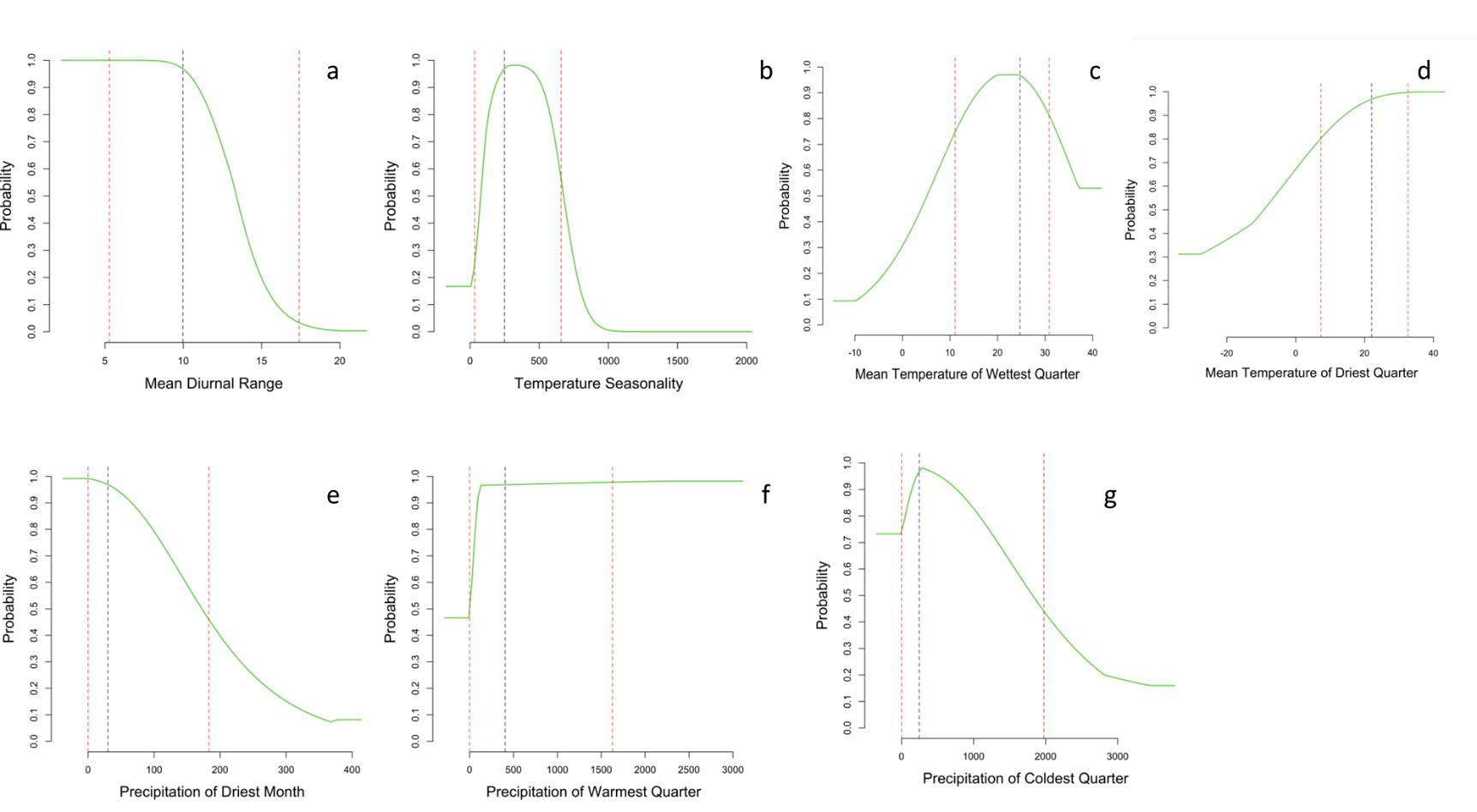


Figure S3. Response curves of the model. a) Bio02 (mean diurnal range), b) Bio04 (temperature seasonality), c) Bio08 (average temperature of the rainy quarter months), d) Bio09 (average temperature of the driest quarter months), e) Bio14 (precipitation of the driest month), f) Bio18 (precipitation of the hottest quarter months), and g) Bio19 (precipitation of the coldest quarter months).


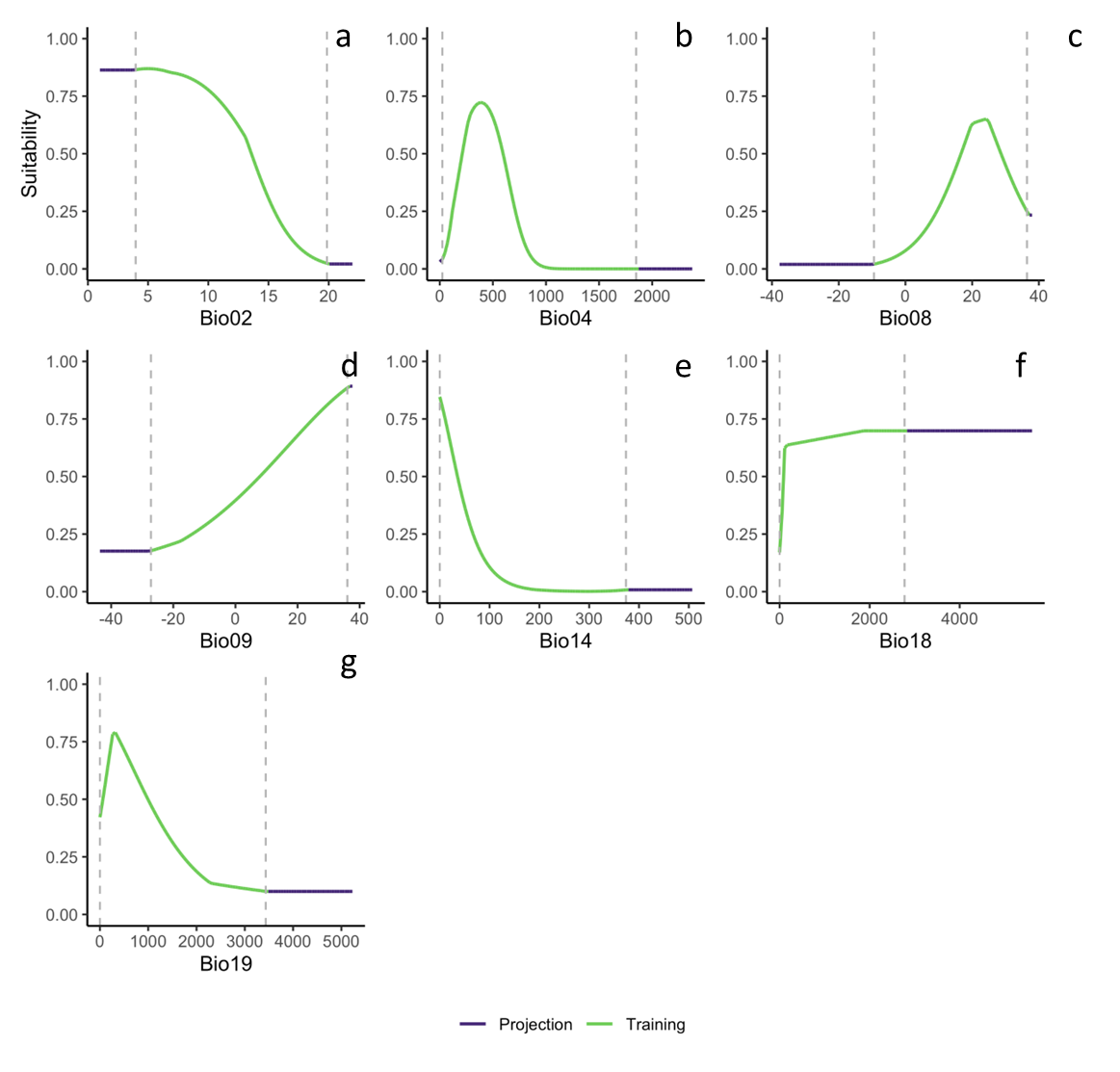


Figure S4. Partial dependence of climatic variables and occurrence of *Sternochetus mangiferae*; a) Bio02 (mean diurnal range), b) Bio04 (temperature seasonality), c) Bio08 (average temperature of the rainy quarter months), d) Bio09 (average temperature of the driest quarter months), e) Bio14 (precipitation of the driest month), f) Bio18 (precipitation of the hottest quarter months), and g) Bio19 (precipitation of the coldest quarter months).


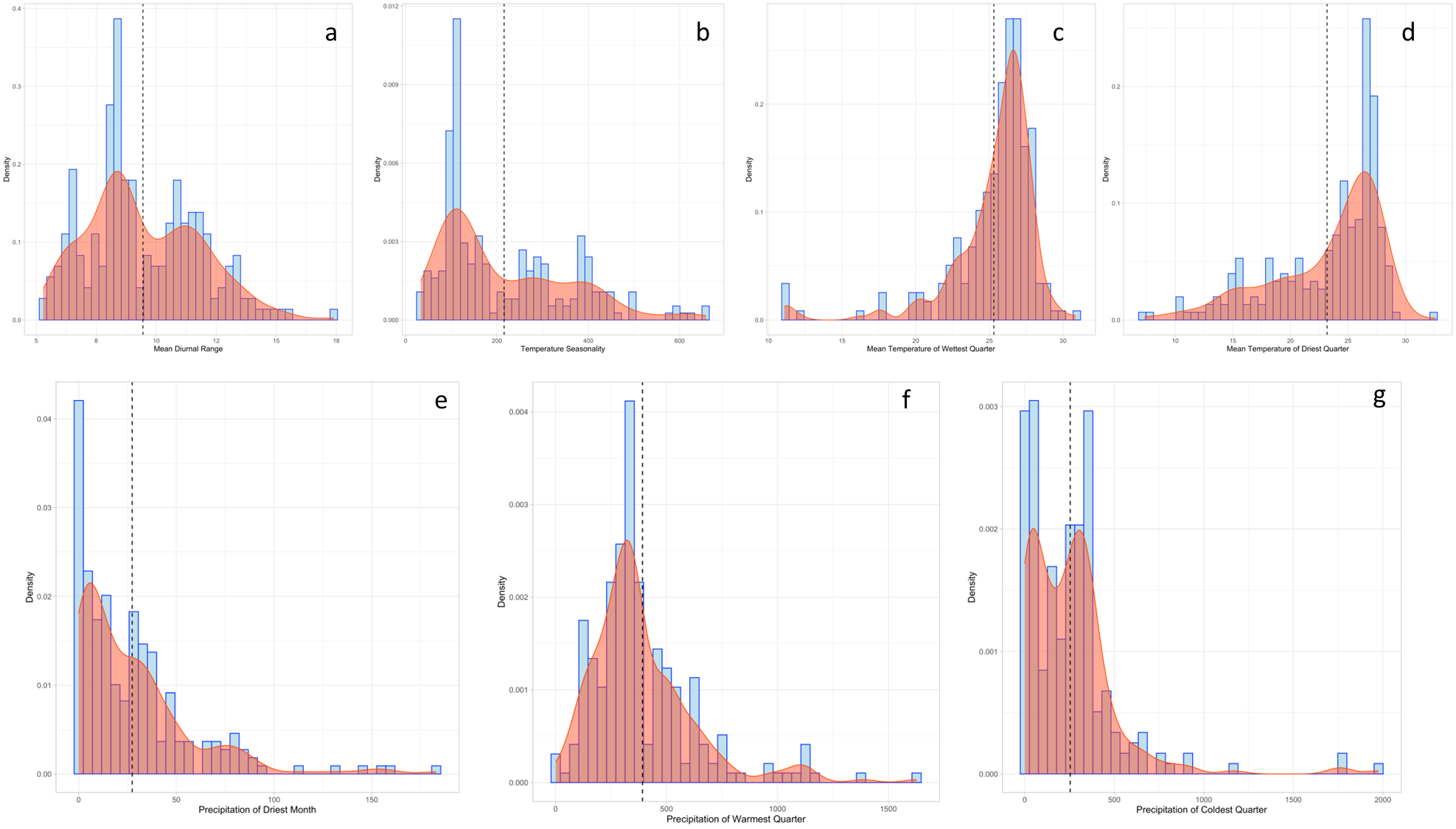


Figure S5. Histogram and Density of occurrence of *Sternochetus mangiferae*) Bio02 (mean diurnal range), b) Bio04 (temperature seasonality), c) Bio08 (average temperature of the rainy quarter months), d) Bio09 (average temperature of the driest quarter months), e) Bio14 (precipitation of the driest month), f) Bio18 (precipitation of the hottest quarter months), and g) Bio19 (precipitation of the coldest quarter months).


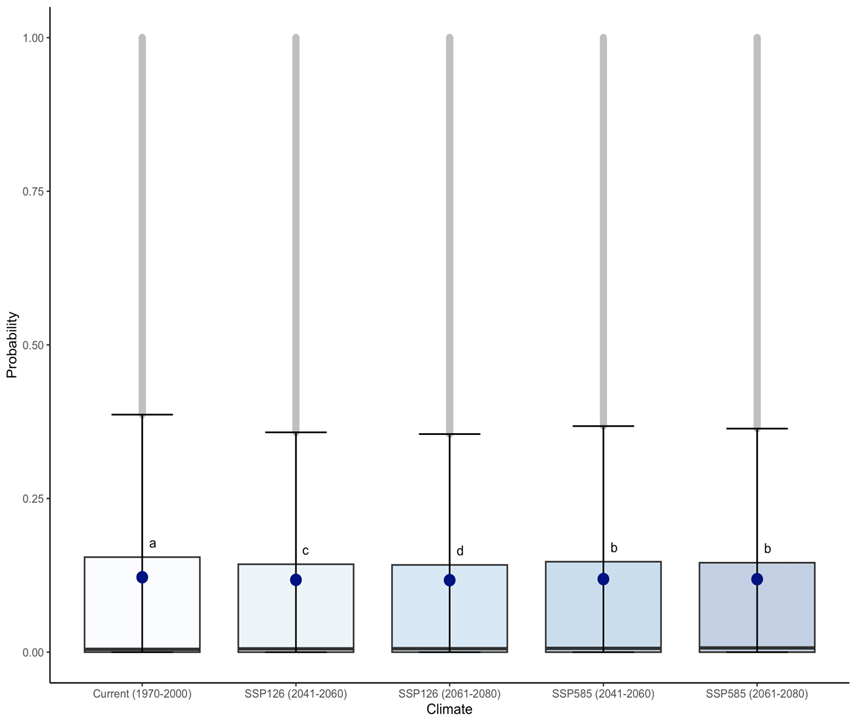


Figure S6. Tukey climates from the current time (1970-2000) to the future (2041-2060; 2061-2080) under SSPs 126 and 585 for the *Sternochetus mangiferae*.
